# Database of glutamate-gated chloride (GluCl) subunits across 125 nematode species: patterns of gene accretion and sequence diversification

**DOI:** 10.1093/g3journal/jkab438

**Published:** 2021-12-21

**Authors:** Damien M O’Halloran

**Affiliations:** Department of Biological Sciences, The George Washington University, Washington, DC 20052, USA

**Keywords:** nematode, glutamate-gated chloride channel, GluCl, database, parasite, anthelmintic, ivermectin

## Abstract

Glutamate-gated chloride channels belong to the Cys-loop receptor superfamily. Glutamate-gated chloride channels are activated by glutamate and form substrates for the antiparasitic drugs from the avermectin family. Glutamate-gated chloride channels are pentameric, and each subunit contains an N-terminal extracellular domain that binds glutamate and 4 helical transmembrane domains, which contain binding sites for avermectin drugs. In order to provide more insight into phylum-wide patterns of glutamate-gated chloride subunit gene expansion and sequence diversity across nematodes, we have developed a database of predicted glutamate-gated chloride subunit genes from 125 nematode species. Our analysis into this dataset described assorted patterns of species-specific glutamate-gated chloride gene counts across different nematodes as well as sequence diversity in key residues thought to be involved in avermectin binding.

## Introduction

Glutamate-gated chloride (GluCl) channels are pentameric ionotropic receptors belonging to the Cys-loop receptor superfamily. Each of the 5 subunits contain an N-terminal extracellular domain that binds Glutamate (Glu) and 4 helical transmembrane domains (TMs). The second TM domain (TM2) from each subunit lines a single channel pore, which opens in response to Glu binding (Hibbs and Gouaux 2011; Althoff et al. 2014). In the free-living nematode *Caenorhabditis elegans* 6 GluCl channel subunit genes have been identified: *glc*-1 to *glc*-4 and *avr*-14 and *avr*-15; while the ruminant parasitic nematode *Haemonchus contortus* has 5 GluCl channel subunit genes: *Hco-glc-2*, *Hco-glc-3*, *Hco-avr-14*, *Hco-glc-5*, and *Hco-glc-6*. GluCl channels are also the substrate for the antiparasitic drug, ivermectin (IVM). IVM is hydrophobic and can reach the GluCl channel TMs to potentiate activity by binding within TM1 and TM2 of adjacent GluCl channel subunits while interacting with residues in the loop between TM2 and TM3 (Cully et al. 1994; Arena et al. 1995; Hibbs and Gouaux 2011; Calimet et al. 2013; Althoff et al. 2014). IVM binding stabilizes the TMs in an open configuration and increases the pore size, thereby increasing channel permeability (Althoff et al. 2014). The genes encoding distinct subunits are modulated differentially by both Glu and IVM: for example, the AVR-15 subunit of *C. elegans* (Dent et al. 1997), and the GluCla2B subunit of *H. contortus* (McCavera et al. 2009) can form homomeric channels that are sensitive to both IVM and Glu, while the *C. elegans* subunit GLC-1 forms homomeric receptors that can be activated by IVM but not by Glu, and conversely homomeric GLC-2 channels can be activated by Glu but not by IVM (Cully et al. 1994; Vassilatis et al. 1997; Daeffler et al. 2014). Interestingly, heteromeric channels comprising GLC-1 and GLC-2 can be activated by both Glu and IVM independently (Cully et al. 1994; Vassilatis et al. 1997; Daeffler et al. 2014). In addition to subunit stoichiometry modulating IVM effects, the sensitivity of GluCl channels to IVM is also modulated by differences in sequence. A naturally occurring 4-amino acid deletion within the ligand-binding domain of GLC-1 in *C. elegans*, has been shown to confer resistance to abamectin, an avermectin class of anthelminthic (Ghosh et al. 2012). IVM resistance was first reported in *H. contortus* in 1979 (van Wyk and Malan 1988), and key residues have been identified within TM3 that are required for IVM sensitivity (Lynagh and Lynch 2010). These findings in *H. contortus* highlight the selection potential placed on GluCl subunit genes in response to IVM treatment, and more GluCl gene sequence data are required to understand population and phylum-wide patterns of sequence diversity. To get at this question of phylum-wide GluCl sequence diversity, we have developed a database of predicted GluCl subunit genes from 125 nematode species. All of these data are freely available for users to search and explore at the following URL: http://ohalloranlab.net/nematode_glucl. Our analysis into this dataset described heterogeneous patterns of species-specific GluCl gene counts across different nematodes as well as sequence diversity in putative substrate residues for IVM binding.

## Materials and methods

Genomes used in this study were obtained from WormBase ParaSite (ver. WBPS15) (Howe et al. 2017). GluCl subunits were predicted from each genome using hidden Markov models (HMMs) as described previously (Langeland et al. 2020, 2021; Wheeler et al. 2020; Bode and O’Halloran 2021). The Pfam (Finn et al. 2014) HMM, PF02932 (*Neur_chan_memb*), was used alongside an HMM based on known GluCl proteins that was generated using *hmmbuild* (Mistry et al. 2013). Next, *hmmsearch* (-*tblout* -*noali*) (Mistry et al. 2013) was used to compare HMMs against genomes obtained from the WormBase Parasite ftp site at the European Bioinformatics Institute (EBI: http://ftp.ebi.ac.uk/pub/databases/wormbase/parasite/releases/WBPS15/species). This approach yielded 983 predicted GluCl subunit genes across all 125 nematode species (Supplementary Table 1). From this starting dataset, alternative splice-forms were removed to get a more accurate gene count per species. After filtering, 864 GluCl subunit genes remained in the dataset and were used as queries in *blastp* (Camacho et al. 2009) searches against a database of characterized GluCl subunits from *C.elegans* and *H.contortus* so as to confirm GluCl identity. Predicted GluCl subunits were then aligned using MAFFT ver. 7.487 (Katoh et al. 2019). Phylogenetic relationships for the aligned orthologous clusters were then reconstructed using PhyML (Guindon et al. 2010) by employing an appropriate model of selection determined using ProtTest3 (Darriba et al. 2011). Heatmaps and trees were rendered and annotated using R (ver. 4.0.5) *pheatmap* package (ver. 1.0.12) (Kolde 2019), *ggtree* package (ver. 2.4.2) (Yu et al. 2017), and *ggtreeExtra* (ver. 1.0.4) (Xu et al. 2021). Orthologous clusters that were refined using this phylogenetic approach were then used to make a database (*makeblastdb*) which was interrogated using local *blast* executables (Camacho et al. 2009) in order to assign orthology to divergent sequences as well as partial sequences from within our database that lacked a clear ortholog. Statistics were performed using Python (ver. 3.9.6) *scipy* module (ver. 1.7.0) and R ver. 4.1.1.

## Results and discussion

**Fig. 1.**
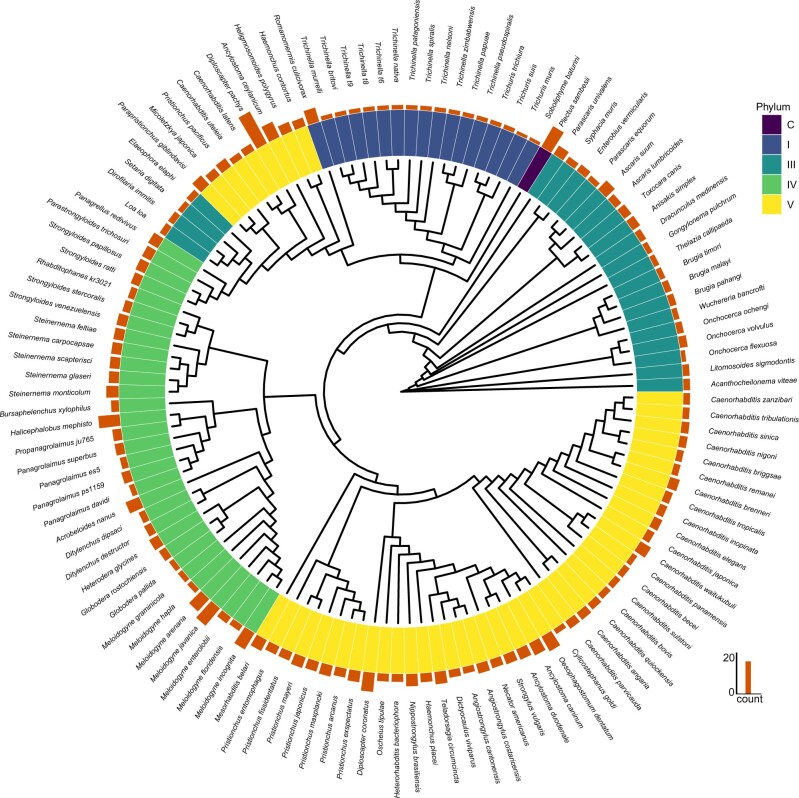
Radial tree representing the total predicted GluCl gene count per species (bar plot) and the corresponding phylum (interior color annotation). The phylogenetic relationship between each species was based on the *rpb-1* gene. Alignments were made using MAFFT (Katoh et al. 2019) while ProtTest 3 (Darriba et al. 2011) was used to determine the best model of selection, which was implemented within PhyML (Guindon et al. 2010) to reconstruct the phylogenetic relationship. Rendering and annotation of the tree was done using the *ggtree* (Yu et al. 2017) and *ggtreeExtra* (ver. 1.0.4) (Xu et al. 2021) packages.

In order to predict GluCl subunit genes, our analyses included genomes from 125 nematode species (Fig. 1; Supplementary Table 1) that covered clades I (17 species), III (24 species), IV (32 species), V (51 species), and C (*Plectus sambesii*), and from this analysis, 864 GluCl subunits genes were identified (Fig. 1). To assess whether there were differences between GluCl subunit gene counts across different Clades, we normalized the predicted GluCl gene counts by dividing the total gene count per species by the number of species within each clade and then compared the normalized value with clade number (Supplementary Fig. 1). From this statistical test, we could conclude that our observed GluCl gene counts per clade were not different to what we would expect for each clade (*P* = 0.4918 Chi-Square Goodness-of-fit test; note: this statistical test excluded clade C as it was represented by only 1 species, and instead only included clades I, III, IV, and V). A lack of discrepancy between our observed GluCl gene counts and expected counts does not rule out possible finer resolution differences, and in fact, we did observe differences in GluCl gene counts across individual species: the species with the most predicted GluCl genes were *Diploscapter pachys*, a free-living clade V nematode, and *P.sambesii* a free-living marine nematode within clade C, while the species with the least predicted GluCl genes were *Soboliphyme baturini*, a clade I intestinal parasite of mustelids and *Trichuris muris*, a clade I intestinal parasite of mice (Fig. 1). Next, we tested whether genome *completeness* influenced GluCl gene counts. Benchmarking Universal Single-Copy Orthologs (BUSCO) scores are the standard measure of genome quality. BUSCO scores measure the completeness of an assembly by looking for the presence or absence of highly conserved genes. By comparing BUSCO scores for each species’ genome assembly against predicted GluCl gene counts we did not observe a significant correlation (Supplementary Fig. 2: *R* = 0.0017, *P* = 0.98), suggesting that while genome improvement may alter some of the final GluCl gene counts, genome quality was not a major driver in the total GluCl gene count differences we observed. It is also worth noting that the majority of assemblies that we used to predict GluCl subunit genes have good BUSCO scores (Supplementary Fig. 3, median BUSCO score = 86.2).

**Fig. 2.**
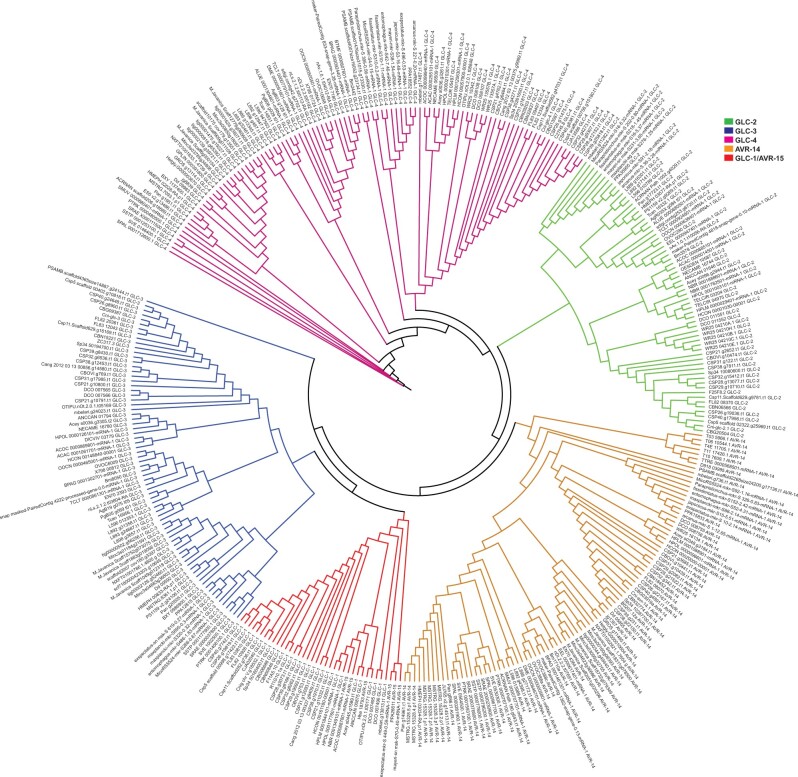
Phylogeny of conserved clusters of predicted GluCl genes inferred using PhyML (Guindon et al. 2010). Sequences clustered into groups of GLC-2, GLC-4, and AVR-14 while GLC-1 and AVR-15 clustered together forming a sister clade to GLC-3.

**Fig. 3.**
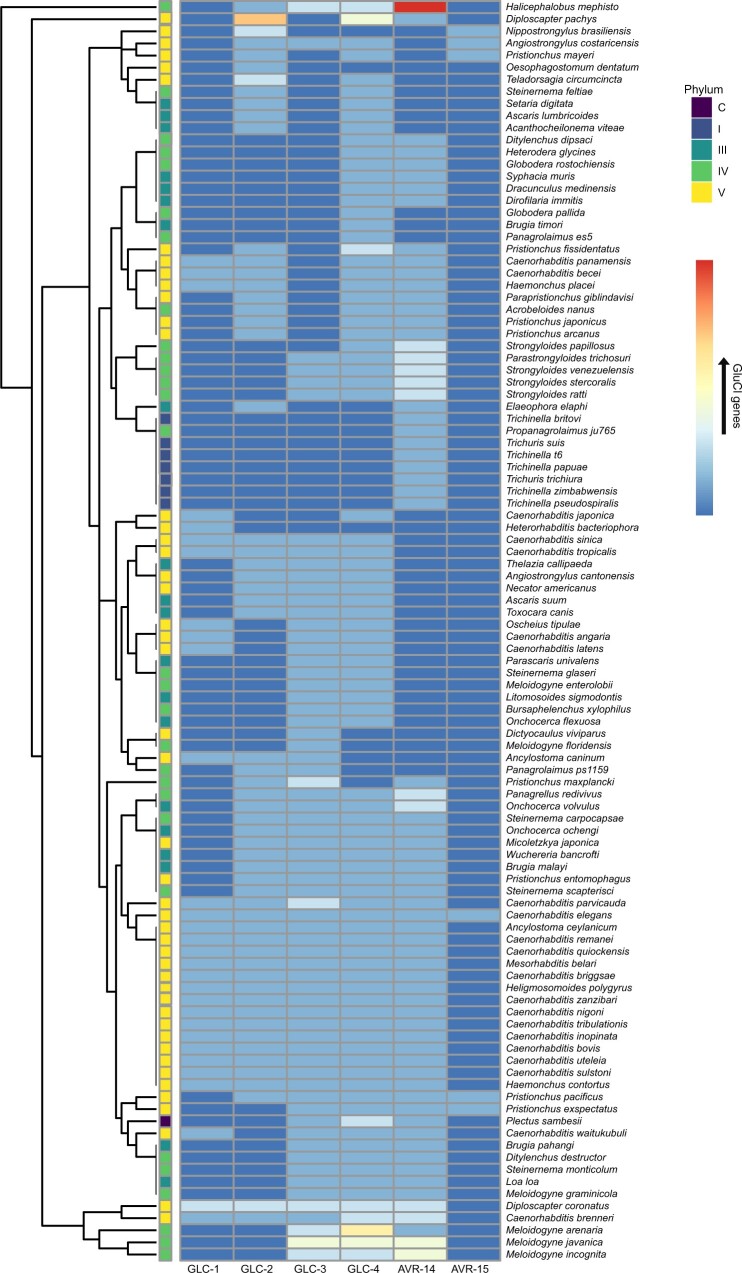
Heatmap of conserved GluCl subunit genes from Fig. 2. Each row corresponds to an individual species while the vertical annotations denote phylum for each species. Hot colors indicate increased predicted GluCl counts while cooler colors represent lower predicted GluCl gene counts.

To organize the 864 GluCl subunits genes that we identified across 125 species into orthologous groups, we performed *blastp* (Camacho et al. 2009) searches of all 864 sequences against a database of characterized GluCl subunits from *C.elegans* and *H.contortus*. This approach revealed clear orthologous clusters for 365 of the GluCl sequences which resulted in *blastp* e-values <5× 10^−324^ (which are typically rounded to 0 via local blastp executables). The classification of these sequences was further tested by examining their phylogenetic relationship; all 365 sequences were aligned using MAFFT (Katoh et al. 2019) and from the alignment the best model of selection was determined to be VT (Müller and Vingron 2000) using ProtTest 3 (Darriba et al. 2011). This information was then used to infer the phylogeny for these conserved 365 GluCl sequences (Fig. 2). The phylogeny revealed that GLC-2, GLC-4, and AVR-14 form separate clades while GLC-1 and AVR-15 both group within the same clade and form a sister clade to GLC-3 (Fig. 2; Supplementary Table 2). These observations suggest that GLC-1 and AVR-15 may represent a more recent duplication event that may postdate a duplication event from GLC-3. This phylogenetic relationship is in keeping with recent observations from another group (Lamassiaude et al. 2021) and is further supported by the synteny of GluCl genes within *C. elegans* in which both GLC-1 and AVR-15 are closely linked on chromosome V while GLC-3 is located toward the other end of chromosome V (Supplementary Fig. 4). Within the conserved group of 365 GluCl sequences that we identified, there was notable expansions within specific species belonging to clades IV and V such as *Halicephalobus mephisto* which has an expansion of predicted *avr-14* subunit genes and also *D.pachys*, which has an expansion of predicted *glc-2* subunit genes (Fig. 3). The *Meloidogyne* species: *M. arenaria*, *M. javanica*, and *M. incognita* displayed expansions of *glc-3*, *glc-4*, and *avr-14* (Fig. 3). It is interesting that 2 species of free-living nematodes as well as 3 plant-parasitic nematode species had specific expansions, and this led us to test whether there were differences between nematode lifestyle (animal parasitic, entomopathogenic, free-living, human parasitic, or plant parasitic) and counts of the predicted GluCl subunit type (*glc-1, glc-2, glc-3, glc-4, avr-14*, or *avr-15*) from our conserved 365 sequences (Figs. 2 and 3). To test this, we normalized the gene counts for each classification group by the number of species within each lifestyle category (Supplementary Fig. 5), and then performed a Chi-Square Goodness-of-fit test as described above for our comparison between clade and GluCl gene counts. From this analysis, we did not observe a significant difference in our observed GluCl gene counts for each lifestyle from what would be expected (*P* = 0.8582), suggesting that predicted GluCl gene expansions in species such as *H. mephisto* and *D. pachys* as well as the *Meloidogyne* species examined may reflect specific lifestyle adaptations.

To classify the remaining 499 GluCl subunit genes from the 864 total GluCl gene predictions into orthologous clusters, we used the conserved 365 predicted GluCl subunit genes that we described above alongside functionally characterized GluCl subunits (Lamassiaude et al. 2021) to create a database which we interrogated using *blastp* searches to cluster into orthologous groups based on top *blastp* hits. These data alongside the conserved 365 predicted GluCl subunit genes were converted into JSON tables to build an online database that included all 864 predicted GluCl sequences. The database is available at the following URL: http://ohalloranlab.net/nematode_GluCl and includes the orthology evidence for each entry (i.e. *phylogeny* for the 365 conserved GluCl subunit genes and *Blast* for the 499 divergent GluCl subunit genes)

The more divergent 499 predicted GluCl sequences that we identified lacked significant conservation across all the sequences which made model selection and phylogenetic reconstructions difficult to interpret. Some of these more divergent predicted GluCl genes exhibited sequence diversity within putative IVM binding sites. While Glu binds the extracellular N-terminal region of GluCl channels, IVM can penetrate to the TM domains and interact with a number of key residues (Hibbs and Gouaux 2011; Althoff et al. 2014). We identified substitutions at a key Glycine residue within TM3 which was mutated to an Alanine within some of the divergent GluCl gene sequences we identified (Supplementary Fig. 6). This Glycine residue is referred to as *M3-Gly* (Lynagh and Lynch 2010), and has been studied in other species to characterize the effect on drug binding. In *H. contortus*, wildtype GluClR α3B exhibited EC_50_ = 39 ± 6 nM, while mutation of M3-Gly to Serine resulted in EC_50_ = 620 ± 140 nM, and mutation of M3-Gly to Alanine caused EC_50_ = 1.2 ± 0.3 µM (Lynagh and Lynch 2010). Interestingly, these mutations also altered Glu sensitivity from wildtype EC_50_ = 15.3 ± 1.8 µM to EC_50_ = 108 ± 10 µM when M3-Gly is mutated to a Serine and EC_50_ = 154 ± 21 µM when M3-Gly is mutated to Alanine (Lynagh and Lynch 2010).

The data described in this paper is available as a searchable database at the following URL: http://ohalloranlab.net/nematode_GluCl. The search textbox at the top of the landing page can be used to query the database for identifiers such as species or GluCl subunit while the columns can be sorted by the clicking the column headings. The raw protein sequence for each entry can be viewed by clicking the green circle in the first cell of a given row, and each entry also links out to WormBase Parasite (Howe et al. 2017) for further exploration. The database can also be searched for similar sequences using local *blast* executables (Camacho et al. 2009) by simply entering a protein sequence into the input field and clicking the submit button to interrogate the nematode GluCl database. We plan to include more species to the database as they become available, and view this database as a platform for users to ask questions related to GluCl evolution and function across diverse nematode species while also providing molecular insights into GluCl pharmacology.

## Supplementary Material

jkab438_Supplemental_Material_Figure_1

jkab438_Supplemental_Material_Figure_2

jkab438_Supplemental_Material_Figure_3

jkab438_Supplemental_Material_Figure_4

jkab438_Supplemental_Material_Figure_5

jkab438_Supplemental_Material_Figure_6

jkab438_Supplemental_Material_Table_1

jkab438_Supplemental_Material_Table_2

jkab438_Supplemental_Material_Legends

## Data Availability

The data underlying this article are available in the article, in its online supplementary material, and at the following URL: http://ohalloranlab.net/nematode_GluCl. Supplemental material is available at *G3* online.
