# Supplementary figures and images for: Database of glutamate-gated chloride (GluCl) subunits across 125 nematode species: patterns of gene accretion and sequence diversification
Source: G3 (Bethesda). 2021 Dec 21;12(2):jkab438. doi: 10.1093/g3journal/jkab438 (PMC9210312; doi:10.1093/g3journal/jkab438)

Figure S1

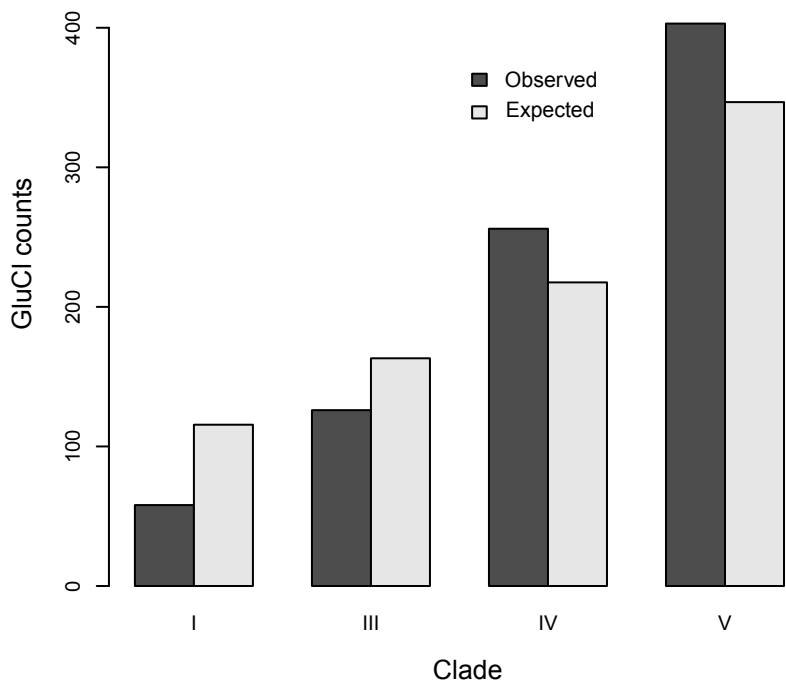

Supplement: jkab438_Supplemental_Material_Figure_1 [file jkab438_supplemental_material_figure_1.pdf]

Figure S2

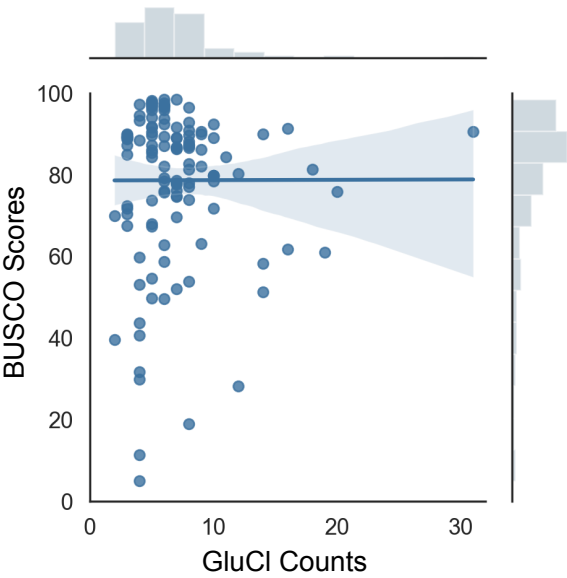

Supplement: jkab438_Supplemental_Material_Figure_2 [file jkab438_supplemental_material_figure_2.pdf]

Figure S3

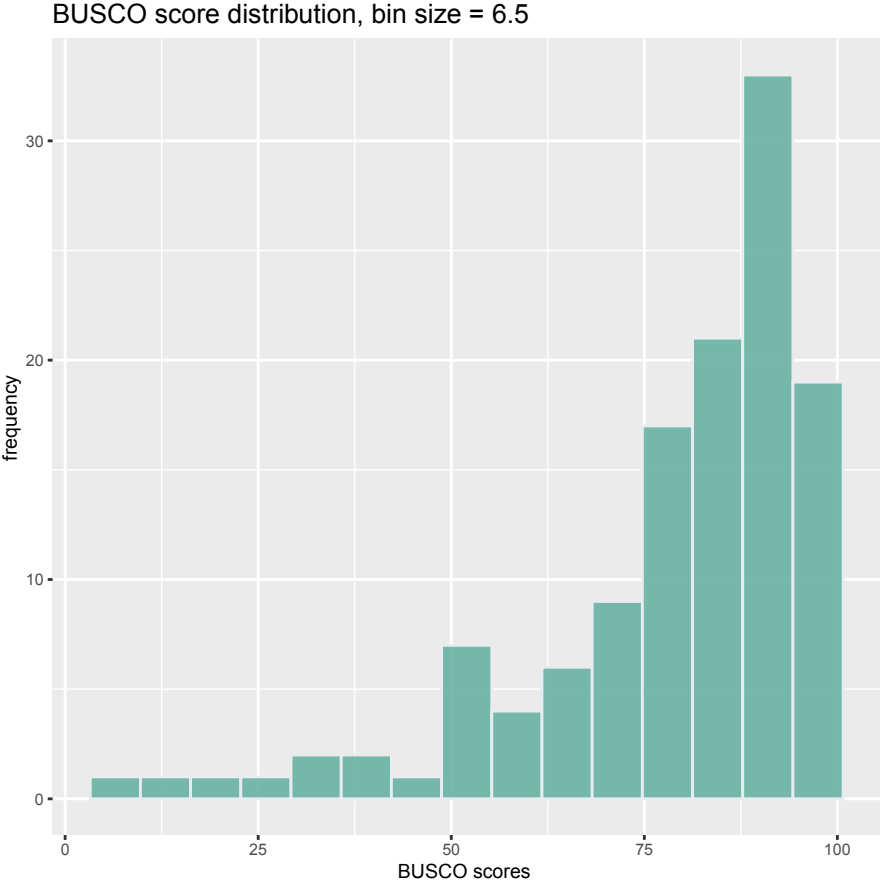

Supplement: jkab438_Supplemental_Material_Figure_3 [file jkab438_supplemental_material_figure_3.pdf]

Figure S4

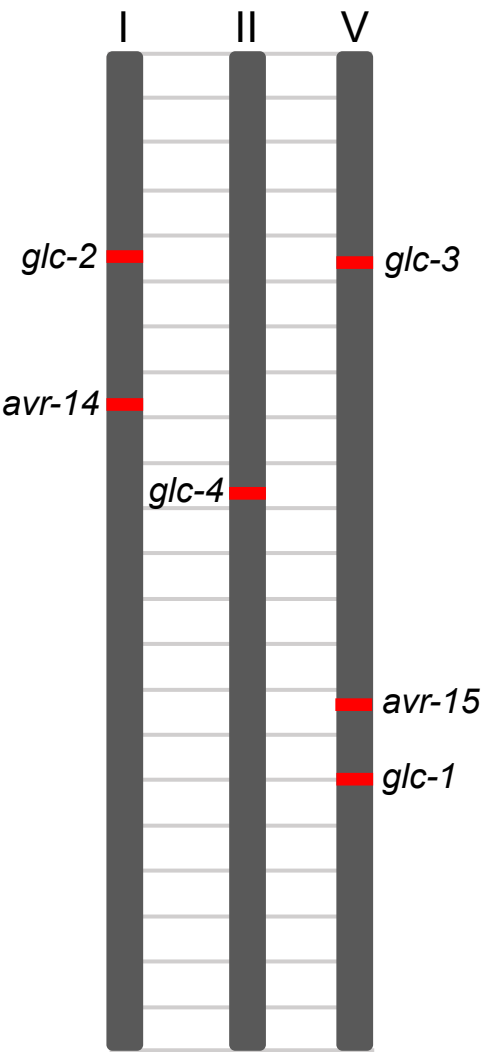

Supplement: jkab438_Supplemental_Material_Figure_4 [file jkab438_supplemental_material_figure_4.pdf]

Figure S5

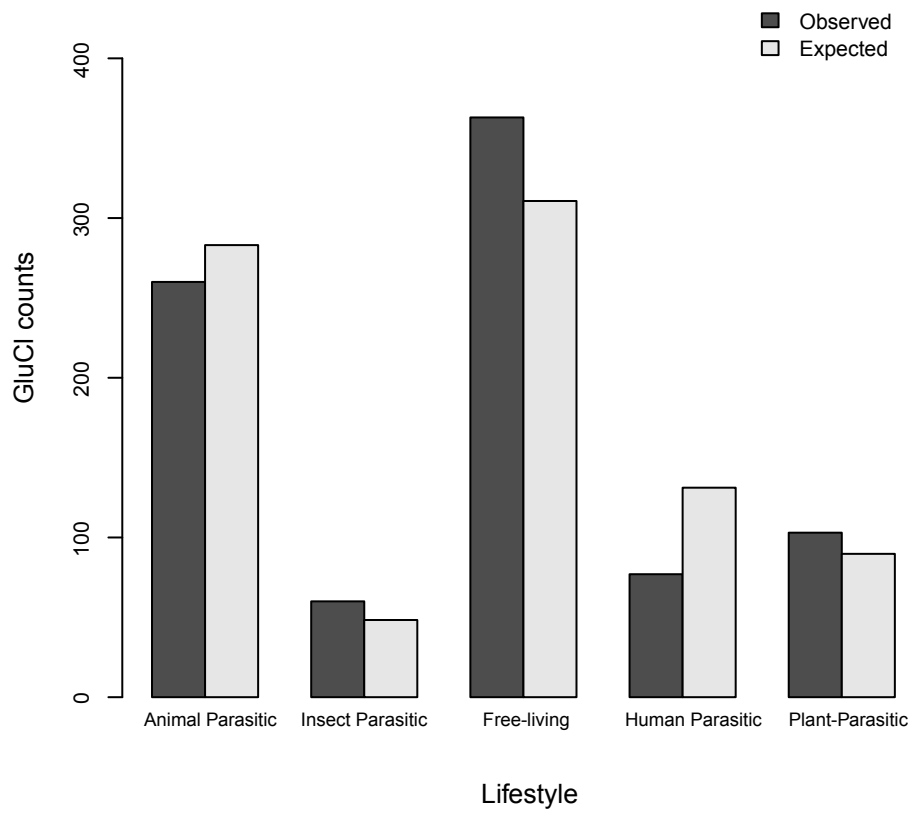

Supplement: jkab438_Supplemental_Material_Figure_5 [file jkab438_supplemental_material_figure_5.pdf]

Figure S6

TM3

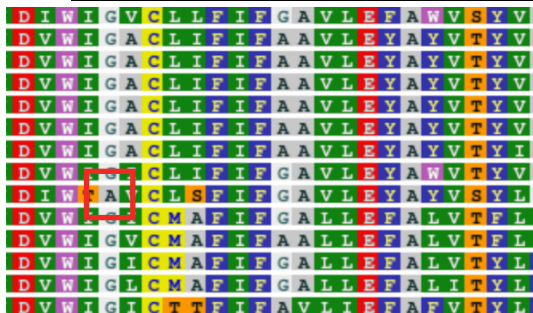

Supplement: jkab438_Supplemental_Material_Figure_6 [file jkab438_supplemental_material_figure_6.pdf]
