# Supplementary material for: Database of glutamate-gated chloride (GluCl) subunits across 125 nematode species: patterns of gene accretion and sequence diversification
Source: G3 (Bethesda). 2021 Dec 21;12(2):jkab438. doi: 10.1093/g3journal/jkab438 (PMC9210312; doi:10.1093/g3journal/jkab438)
Supplement: jkab438_Supplemental_Material_Table_1 [file jkab438_supplemental_material_table_1.docx]

**Table S1.** Species and genome assembly data used to predict GluCl genes

| **Species Name** | **Provider** | **Assembly** | **BioProject** | **Clade** | **N50** | **BUSCO** |
| --- | --- | --- | --- | --- | --- | --- |
| *Acanthocheilonema viteae* | University of Edinburgh | ASM90053725v1 | PRJEB1697 | III | 25,752 | 90.5 |
| *Acrobeloides nanus* | University College London | v1 | PRJEB26554 | IV | 19,572 | 80.4 |
| *Ancylostoma caninum* | McDonnell Genome Institute | A_caninum_9.3.2.ec.cg.pg | PRJNA72585 | V | 255,299 | 84.4 |
| *Ancylostoma ceylanicum* | Cornell University | Acey_2013.11.30.genDNA | PRJNA231479 | V | 668,412 | 90.1 |
| *Ancylostoma duodenale* | McDonnell Genome Institute | A_duodenale_2.2.ec.cg.pg | PRJNA72581 | V | 10,112 | 63.2 |
| *Angiostrongylus cantonensis* | Wellcome Sanger Institute | A_cantonensis_Taipei_v1_5_4 | PRJEB493 | V | 43,712 | 68.1 |
| *Angiostrongylus costaricensis* | Wellcome Sanger Institute | A_costaricensis_Costa_Rica_0011_upd | PRJEB494 | V | 111,998 | 82.7 |
| *Anisakis simplex* | Wellcome Sanger Institute | A_simplex_0011_upd | PRJEB496 | III | 9,229 | 52.1 |
| *Ascaris lumbricoides* | Wellcome Sanger Institute | A_lumbricoides_Ecuador_v1_5_4 | PRJEB4950 | III | 55,909 | 82.2 |
| *Ascaris suum* | University of Colorado Denver | ASM18702v3 | PRJNA62057 | III | 4,646,302 | 89.1 |
| *Brugia malayi* | WormBase | Bmal-4.0 | PRJNA10729 | III | 14,214,749 | 96.7 |
| *Brugia pahangi* | Wellcome Sanger Institute | B_pahangi_Glasgow_0011_upd | PRJEB497 | III | 65,530 | 89.7 |
| *Brugia timori* | Wellcome Sanger Institute | B_timori_Indonesia_v1_0_4_001_upd | PRJEB4663 | III | 4,903 | 53.1 |
| *Bursaphelenchus xylophilus* | Wellcome Sanger Institute | ASM23113v1_submitted | PRJEA64437 | IV | 949,830 | 75.8 |
| *Caenorhabditis angaria* | California Institute of Technology | 13-Mar-12 | PRJNA51225 | V | 79,665 | 77.7 |
| *Caenorhabditis becei* | BANG | CBECE_v1 | PRJEB28243 | V | 767,505 | 97.6 |
| *Caenorhabditis bovis* | BANG | CBOVIS_v1 | PRJEB34497 | V | 7,557,458 | 94.2 |
| *Caenorhabditis brenneri* | WormBase | C_brenneri-6.0.1b | PRJNA20035 | V | 377,250 | 96.6 |
| *Caenorhabditis briggsae* | WormBase | CB4 | PRJNA10731 | V | 17,485,439 | 97.7 |
| *Caenorhabditis elegans* | WormBase | WBcel235 | PRJNA13758 | V | 17,493,829 | 98.6 |
| *Caenorhabditis inopinata* | University of Miyazaki | Sp34_v7 | PRJDB5687 | V | 20,594,552 | 96.6 |
| *Caenorhabditis japonica* | WormBase | C_japonica-7.0.1 | PRJNA12591 | V | 94,114 | 92.4 |
| *Caenorhabditis latens* | University of Oregon | CaeLat1.0 | PRJNA248912 | V | 366,678 | 97 |
| *Caenorhabditis nigoni* | Cornell University | nigoni.pc_2016.07.14 | PRJNA384657 | V | 20,390,332 | 98.5 |
| *Caenorhabditis panamensis* | BANG | CPANA_v1 | PRJEB28259 | V | 487,150 | 97.3 |
| *Caenorhabditis parvicauda* | BANG | CPARV_v1 | PRJEB12595 | V | 44,393 | 89.2 |
| *Caenorhabditis quiockensis* | BANG | CQUIO_v1 | PRJEB11354 | V | 139,266 | 95.9 |
| *Caenorhabditis remanei* | University of Oregon | CaeRem1.0 | PRJNA248911 | V | 1,765,890 | 97 |
| *Caenorhabditis sinica* | University of Edinburgh | 1 | PRJNA194557 | V | 25,151 | 93.8 |
| *Caenorhabditis sulstoni* | BANG | CSULS_v1 | PRJEB12601 | V | 136,669 | 97.9 |
| *Caenorhabditis tribulationis* | BANG | CTRIB_v1 | PRJEB12608 | V | 224,519 | 97.7 |
| *Caenorhabditis tropicalis* | McDonnell Genome Institute | Caenorhabditis_sp11_JU1373-3.0.1 | PRJNA53597 | V | 20,921,866 | 97 |
| *Caenorhabditis uteleia* | BANG | CUTEL_v1 | PRJEB12600 | V | 175,547 | 96 |
| *Caenorhabditis waitukubuli* | BANG | CWAIT_v1 | PRJEB12602 | V | 15,045 | 92.5 |
| *Caenorhabditis zanzibari* | BANG | CZANZ_v1 | PRJEB12596 | V | 91,045 | 98.2 |
| *Cylicostephanus goldi* | Wellcome Sanger Institute | C_goldi_Cheshire_0011 | PRJEB498 | V | 1,235 | 11.4 |
| *Dictyocaulus viviparus* | McDonnell Genome Institute | D_viviparus_9.2.1.ec.pg | PRJNA72587 | V | 225,234 | 62.9 |
| *Diploscapter coronatus* | National Institute of Genetics, Japan | ASM220778v1 | PRJDB3143 | V | 1,007,652 | 91.4 |
| *Diploscapter pachys* | New York University | DipSp1Ass11Ann3 | PRJNA280107 | V | 124,169 | 90.7 |
| *Dirofilaria immitis* | University of Edinburgh | nDi.2.2 | PRJEB1797 | III | 71,050 | 91.7 |
| *Ditylenchus destructor* | Huazhong Agricultural University | ASM157970v1 | PRJNA312427 | IV | 555,026 | 76.1 |
| *Ditylenchus dipsaci* | Agriculture and Agri-Food Canada | D.dipsaci.v1.0 | PRJNA498219 | IV | 287,009 | 54.7 |
| *Dracunculus medinensis* | Wellcome Sanger Institute | D_medinensis_Ghana_v2_0_4 | PRJEB500 | III | 643,251 | 86.1 |
| *Elaeophora elaphi* | Wellcome Sanger Institute | E_elaphi_v1_0_4 | PRJEB502 | III | 25,422 | 87.3 |
| *Enterobius vermicularis* | Wellcome Sanger Institute | E_vermicularis_Canary_Islands_upd | PRJEB503 | III | 20,546 | 79.3 |
| *Globodera pallida* | Wellcome Sanger Institute | GPAL001 | PRJEB123 | IV | 120,163 | 43.7 |
| *Globodera rostochiensis* | University of Edinburgh | nGr | PRJEB13504 | IV | 88,495 | 58.8 |
| *Gongylonema pulchrum* | Wellcome Sanger Institute | G_pulchrum_Hokkaido_0011_upd | PRJEB505 | III | 4,421 | 31.8 |
| *Haemonchus contortus* | Wellcome Sanger Institute | haemonchus_contortus_MHCO3ISE_4.0 | PRJEB506 | V | 47,382,676 | 86.5 |
| *Haemonchus placei* | Wellcome Sanger Institute | H_placei_MHpl1_0011_upd | PRJEB509 | V | 37,562 | 78.7 |
| *Halicephalobus mephisto* | American University, Washington, D.C. | SWDT01000000 | PRJNA528747 | IV | 313,311 | 81.4 |
| *Heligmosomoides polygyrus* | University of Edinburgh | nHp_v2.0 | PRJEB15396 | V | 179,069 | 87.5 |
| *Heterodera glycines* | Iowa State University | Hetgly_1 | PRJNA381081 | IV | 304,127 | 54 |
| *Heterorhabditis bacteriophora* | McDonnell Genome Institute | Heterorhabditis_bacteriophora-7.0 | PRJNA13977 | V | 312,328 | 87.1 |
| *Litomosoides sigmodontis* | University of Edinburgh | ASM90053727v1 | PRJEB3075 | III | 45,733 | 91.8 |
| *Loa loa* | Institute for Genome Sciences | LLoa-hgap-1 | PRJNA246086 | III | 180,288 | 97.5 |
| *Meloidogyne arenaria* | RIKEN | ASM313380v1 | PRJNA438575 | IV | 204,341 | 58.4 |
| *Meloidogyne graminicola* | IARI | Mgraminicola_V1 | PRJNA411966 | IV | 20,427 | 49.7 |
| *Meloidogyne hapla* | North Carolina State University | Freeze_1 | PRJNA29083 | IV | 37,501 | 49.9 |
| *Meloidogyne javanica* | University of Hull | ASM369362v1 | PRJNA340324 | IV | 14,103 | 40.7 |
| *Meloidogyne javanica* | University of Hull | ASM369362v1 | PRJNA340324 | IV | 14,103 | 59.9 |
| *Meloidogyne javanica* | INRA | ASM90000394v1 | PRJEB8714 | IV | 10,373 | 61.8 |
| *Meloidogyne javanica* | INRA | ASM90000394v1 | PRJEB8714 | IV | 10,373 | 61.1 |
| *Mesorhabditis belari* | ENS Lyon | M.Belari_Hybrid_genome | PRJEB30104 | V | 462,517 | 79.9 |
| *Necator americanus* | McDonnell Genome Institute | N__americanus_v1 | PRJNA72135 | V | 211,860 | 86.4 |
| *Nippostrongylus brasiliensis* | Wellcome Sanger Institute | N_brasiliensis_RM07_v1_5_4_0011_upd | PRJEB511 | V | 33,446 | 76.3 |
| *Oesophagostomum dentatum* | McDonnell Genome Institute | O_dentatum_10.0.ec.cg.pg | PRJNA72579 | V | 19,257 | 79.9 |
| *Onchocerca flexuosa* | McDonnell Genome Institute | O_flexuosa_1.0.allpaths.pg.lrna | PRJNA230512 | III | 540,294 | 51.3 |
| *Onchocerca ochengi* | Wellcome Sanger Institute | O_ochengi_Ngaoundere | PRJEB1204 | III | 16,140 | 72.4 |
| *Onchocerca volvulus* | WormBase | ASM49940v2 | PRJEB513 | III | 25,485,961 | 85.5 |
| *Oscheius tipulae* | University of Edinburgh | Oscheius_tipulae_assembly_v2 | PRJEB15512 | V | 1,203,411 | 97.6 |
| *Panagrellus redivivus* | California Institute of Technology | Pred3 | PRJNA186477 | IV | 262,414 | 89.4 |
| *Parascaris equorum* | Wellcome Sanger Institute | P_equorum_v1_0_4 | PRJEB514 | III | 5,333 | 82.1 |
| *Parascaris univalens* | University of Colorado Denver | ASM225920v1 | PRJNA386823 | III | 1,825,986 | 30 |
| *Parastrongyloides trichosuri* | Wellcome Sanger Institute | P_trichosuri_KNP | PRJEB515 | IV | 836,942 | 67.4 |
| *Plectus sambesii* | MRC London Institute of Medical Sciences | Psam_v1.0 | PRJNA390260 | C | 23,404 | 74.7 |
| *Pristionchus exspectatus* | MPIfor Developmental Biology | Pristionchus_exspectatus_de_novo_assembly | PRJEB24288 | V | 142,175 | 69.7 |
| *Pristionchus mayeri* | MPIfor Developmental Biology | Pristionchus_mayeri_genome | PRJEB27334 | V | 234,324 | 78.5 |
| *Pristionchus mayeri* | MPIfor Developmental Biology | Pristionchus_mayeri_genome | PRJEB27334 | V | 234,324 | 5 |
| *Pristionchus mayeri* | MPIfor Developmental Biology | Pristionchus_mayeri_genome | PRJEB27334 | V | 234,324 | 90.9 |
| *Pristionchus mayeri* | MPIfor Developmental Biology | Pristionchus_mayeri_genome | PRJEB27334 | V | 234,324 | 78.1 |
| *Pristionchus mayeri* | MPIfor Developmental Biology | Pristionchus_mayeri_genome | PRJEB27334 | V | 234,324 | 75.9 |
| *Pristionchus mayeri* | MPIfor Developmental Biology | Pristionchus_mayeri_genome | PRJEB27334 | V | 234,324 | 91.7 |
| *Pristionchus mayeri* | MPIfor Developmental Biology | Pristionchus_mayeri_genome | PRJEB27334 | V | 234,324 | 86.9 |
| *Pristionchus mayeri* | MPIfor Developmental Biology | Pristionchus_mayeri_genome | PRJEB27334 | V | 234,324 | 90.6 |
| *Pristionchus pacificus* | WormBase | El_Paco | PRJNA12644 | V | 23,915,096 | 89.1 |
| *Propanagrolaimus ju765* | University College London | JU765_v2 | PRJEB32708 | IV | 10,830 | 89.8 |
| *Propanagrolaimus ju765* | University College London | JU765_v2 | PRJEB32708 | IV | 10,830 | 90 |
| *Propanagrolaimus ju765* | University College London | JU765_v2 | PRJEB32708 | IV | 10,830 | 86.3 |
| *Propanagrolaimus ju765* | University College London | JU765_v2 | PRJEB32708 | IV | 10,830 | 92.9 |
| *Propanagrolaimus ju765* | University College London | JU765_v2 | PRJEB32708 | IV | 10,830 | 74.8 |
| *Rhabditophanes sp. KR3021* | Wellcome Sanger Institute | Rhabditophanes_sp_KR3021 | PRJEB1297 | IV | 537,195 | 73.8 |
| *Romanomermis culicivorax* | University of Cologne | nRc.2.0 | PRJEB1358 | I | 17,583 | 28.3 |
| *Setaria digitata* | University of Colombo | ASM364038v1 | PRJNA479729 | III | 120,860 | 94.6 |
| *Soboliphyme baturini* | Wellcome Sanger Institute | S_baturini_Dall_Island_0011_upd | PRJEB516 | I | 19,742 | 39.6 |
| *Steinernema carpocapsae* | University of California, Irvine | S_carpo_v1_submitted | PRJNA202318 | IV | 299,566 | 87.1 |
| *Steinernema feltiae* | California Institute of Technology | S_felt_v1_submitted | PRJNA204661 | IV | 47,472 | 86.7 |
| *Steinernema glaseri* | California Institute of Technology | S_glas_v1_submitted | PRJNA204943 | IV | 37,382 | 81.4 |
| *Steinernema monticolum* | California Institute of Technology | S_monti_v1_submitted | PRJNA205067 | IV | 11,533 | 79.9 |
| *Steinernema scapterisci* | California Institute of Technology | S_scapt_v1_submitted | PRJNA204942 | IV | 90,783 | 88.3 |
| *Strongyloides papillosus* | Wellcome Sanger Institute | S_papillosus_LIN_v2_1_4 | PRJEB525 | IV | 86,067 | 77.1 |
| *Strongyloides ratti* | Wellcome Sanger Institute | S_ratti_ED321_v5_0_4 | PRJEB125 | IV | 11,693,564 | 78.1 |
| *Strongyloides stercoralis* | Wellcome Sanger Institute | S_stercoralis_PV0001_v2_0_4 | PRJEB528 | IV | 431,128 | 78.5 |
| *Strongyloides venezuelensis* | Wellcome Sanger Institute | S_venezuelensis_HH1 | PRJEB530 | IV | 715,404 | 74 |
| *Strongylus vulgaris* | Wellcome Sanger Institute | S_vulgaris_Kentucky_0011_upd | PRJEB531 | V | 2,377 | 19 |
| *Syphacia muris* | Wellcome Sanger Institute | S_muris_Valencia_v1_0_4 | PRJEB524 | III | 60,614 | 84.4 |
| *Teladorsagia circumcincta* | McDonnell Genome Institute | T_circumcincta.14.0.ec.cg.pg | PRJNA72569 | V | 46,814 | 71.9 |
| *Thelazia callipaeda* | Wellcome Sanger Institute | T_callipaeda_Ticino_0011_upd | PRJEB1205 | III | 50,894 | 88.6 |
| *Toxocara canis* | University of Melbourne | Toxocara_canis_adult_r1.0 | PRJNA248777 | III | 374,962 | 86.7 |
| *Trichinella spiralis* | McDonnell Genome Institute | Trichinella_spiralis-3.7.1 | PRJNA12603 | I | 6,373,445 | 89.4 |
| *Trichinella zimbabwensis* | University of Melbourne | T11_ISS1029_r1.0 | PRJNA257433 | I | 205,645 | 88.8 |
| *Trichinella zimbabwensis* | University of Melbourne | T11_ISS1029_r1.0 | PRJNA257433 | I | 205,645 | 90 |
| *Trichinella zimbabwensis* | University of Melbourne | T11_ISS1029_r1.0 | PRJNA257433 | I | 205,645 | 89.2 |
| *Trichinella zimbabwensis* | University of Melbourne | T11_ISS1029_r1.0 | PRJNA257433 | I | 205,645 | 71.8 |
| *Trichinella zimbabwensis* | University of Melbourne | T11_ISS1029_r1.0 | PRJNA257433 | I | 205,645 | 89.9 |
| *Trichinella zimbabwensis* | University of Melbourne | T11_ISS1029_r1.0 | PRJNA257433 | I | 205,645 | 85.1 |
| *Trichinella zimbabwensis* | University of Melbourne | T11_ISS1029_r1.0 | PRJNA257433 | I | 205,645 | 89.3 |
| *Trichinella zimbabwensis* | University of Melbourne | T11_ISS1029_r1.0 | PRJNA257433 | I | 205,645 | 89.5 |
| *Trichinella zimbabwensis* | University of Melbourne | T11_ISS1029_r1.0 | PRJNA257433 | I | 205,645 | 89.6 |
| *Trichinella zimbabwensis* | University of Melbourne | T11_ISS1029_r1.0 | PRJNA257433 | I | 205,645 | 89.2 |
| *Trichinella zimbabwensis* | University of Melbourne | T11_ISS1029_r1.0 | PRJNA257433 | I | 205,645 | 87.3 |
| *Trichuris muris* | WormBase | TMUE3.0 | PRJEB126 | I | 28,941,788 | 70 |
| *Trichuris suis* | McDonnell Genome Institute | T_suis_1.0.allpaths | PRJNA179528 | I | 1,322,386 | 70.4 |
| *Trichuris trichiura* | Wellcome Sanger Institute | TTRE2.1 | PRJEB535 | I | 69,794 | 67.6 |
| *Wuchereria bancrofti* | Case Western Reserve University | Wb_PNG_Genome_assembly_pt22 | PRJNA275548 | III | 56,656 | 93.4 |
