# Supplementary material for: Database of glutamate-gated chloride (GluCl) subunits across 125 nematode species: patterns of gene accretion and sequence diversification
Source: G3 (Bethesda). 2021 Dec 21;12(2):jkab438. doi: 10.1093/g3journal/jkab438 (PMC9210312; doi:10.1093/g3journal/jkab438)
Supplement: jkab438_Supplemental_Material_Table_2 [file jkab438_supplemental_material_table_2.docx]

| **Species** | **Transcript** |
| --- | --- |
| *Angiostrongylus cantonensis* | ACAC_0000014501-mRNA-1 |
| *Angiostrongylus cantonensis* | ACAC_0000255101-mRNA-1 |
| *Angiostrongylus cantonensis* | ACAC_0001061701-mRNA-1 |
| *Ancylostoma ceylanicum* | Acey_s0036.g3261.t1 |
| *Ancylostoma ceylanicum* | Acey_s0036.g3305.t2 |
| *Ancylostoma ceylanicum* | Acey_s0096.g2944.t1 |
| *Ancylostoma ceylanicum* | Acey_s0348.g3184.t1 |
| *Ancylostoma ceylanicum* | Acey_s0445.g1589.t1 |
| *Angiostrongylus costaricensis* | ACOC_0000869801-mRNA-1 |
| *Angiostrongylus costaricensis* | ACOC_0000885101-mRNA-1 |
| *Angiostrongylus costaricensis* | ACOC_0000887801-mRNA-1 |
| *Angiostrongylus costaricensis* | ACOC_0000999401-mRNA-1 |
| *Acrobeloides nanus* | ACRNAN_Path_1052.g4029.t1 |
| *Acrobeloides nanus* | ACRNAN_Path_180.g643.t1 |
| *Acrobeloides nanus* | ACRNAN_scaffold206.g18669.t1 |
| *Ascaris suum* | AgB03_g128_t01 |
| *Ascaris suum* | AgB19_g075_t05 |
| *Ascaris suum* | AgR035X_g084_t01 |
| *Ascaris lumbricoides* | ALUE_0000680501-mRNA-1 |
| *Ascaris lumbricoides* | ALUE_0001304801-mRNA-1 |
| *Ancylostoma caninum* | ANCCAN_00875 |
| *Ancylostoma caninum* | ANCCAN_01646 |
| *Ancylostoma caninum* | ANCCAN_01794 |
| *Pristionchus arcanus* | arcanus-mkr-S_1-12.65-mRNA-1 |
| *Pristionchus arcanus* | arcanus-mkr-S_221-0.20-mRNA-1 |
| *Pristionchus arcanus* | arcanus-mkr-S_36-3.28-mRNA-1 |
| *Caenorhabditis elegans* | B0207.12a |
| *Brugia malayi* | Bm1710a |
| *Brugia malayi* | Bm2442 |
| *Brugia malayi* | Bm3578 |
| *Brugia malayi* | Bm8050 |
| *Brugia pahangi* | BPAG_0000924401-mRNA-1 |
| *Brugia pahangi* | BPAG_0001302701-mRNA-1 |
| *Brugia pahangi* | BPAG_0001461301-mRNA-1 |
| *Brugia timori* | BTMF_0000807601-mRNA-1 |
| *Bursaphelenchus xylophilus* | BXY_0986900.1 |
| *Bursaphelenchus xylophilus* | BXY_1137400.1 |
| *Caenorhabditis elegans* | C27H5.8 |
| *Caenorhabditis angaria* | Cang_2012_03_13_00337.g9409.t1 |
| *Caenorhabditis angaria* | Cang_2012_03_13_00375.g9969.t1 |
| *Caenorhabditis angaria* | Cang_2012_03_13_00856.g14580.t1 |
| *Caenorhabditis briggsae* | CBG03919a |
| *Caenorhabditis briggsae* | CBG06688 |
| *Caenorhabditis briggsae* | CBG09387 |
| *Caenorhabditis briggsae* | CBG13087 |
| *Caenorhabditis briggsae* | CBG20504 |
| *Caenorhabditis brenneri* | CBN00846 |
| *Caenorhabditis brenneri* | CBN06566 |
| *Caenorhabditis brenneri* | CBN15221 |
| *Caenorhabditis brenneri* | CBN18452 |
| *Caenorhabditis brenneri* | CBN29203 |
| *Caenorhabditis brenneri* | CBN32774 |
| *Caenorhabditis brenneri* | CBN32837 |
| *Caenorhabditis bovis* | CBOVI.g10441.t1 |
| *Caenorhabditis bovis* | CBOVI.g10474.t1 |
| *Caenorhabditis bovis* | CBOVI.g6464.t1 |
| *Caenorhabditis bovis* | CBOVI.g753.t1 |
| *Caenorhabditis bovis* | CBOVI.g769.t1 |
| *Caenorhabditis japonica* | CJA02040 |
| *Caenorhabditis japonica* | CJA13586 |
| *Caenorhabditis nigoni* | Cni-avr-14.1 |
| *Caenorhabditis nigoni* | Cni-glc-2.1 |
| *Caenorhabditis nigoni* | Cni-glc-3 |
| *Caenorhabditis nigoni* | Cni-glc-4.1 |
| *Caenorhabditis nigoni* | Cnig_chr_V.g18532.1 |
| *Caenorhabditis tropicalis* | Csp11.Scaffold282.g736.t1 |
| *Caenorhabditis tropicalis* | Csp11.Scaffold582.g4703.t1 |
| *Caenorhabditis tropicalis* | Csp11.Scaffold629.g16169.t1 |
| *Caenorhabditis tropicalis* | Csp11.Scaffold629.g9781.t1 |
| *Caenorhabditis parvicauda* | CSP21.g10791.t1 |
| *Caenorhabditis parvicauda* | CSP21.g10800.t1 |
| *Caenorhabditis parvicauda* | CSP21.g11032.t1 |
| *Caenorhabditis parvicauda* | CSP21.g2652.t1 |
| *Caenorhabditis parvicauda* | CSP21.g3685.t1 |
| *Caenorhabditis parvicauda* | CSP21.g4762.t1 |
| *Caenorhabditis zanzibari* | CSP26.g11475.t1 |
| *Caenorhabditis zanzibari* | CSP26.g19036.t1 |
| *Caenorhabditis zanzibari* | CSP26.g19819.t1 |
| *Caenorhabditis zanzibari* | CSP26.g22167.t1 |
| *Caenorhabditis zanzibari* | CSP26.g8900.t1 |
| *Caenorhabditis panamensis* | CSP28.g12152.t1 |
| *Caenorhabditis panamensis* | CSP28.g13077.t1 |
| *Caenorhabditis panamensis* | CSP28.g21522.t1 |
| *Caenorhabditis panamensis* | CSP28.g3073.t1 |
| *Caenorhabditis becei* | CSP29.g10710.t1 |
| *Caenorhabditis becei* | CSP29.g19092.t1 |
| *Caenorhabditis becei* | CSP29.g2082.t1 |
| *Caenorhabditis becei* | CSP29.g9307.t1 |
| *Caenorhabditis uteleia* | CSP31.g10439.t1 |
| *Caenorhabditis uteleia* | CSP31.g12093.t1 |
| *Caenorhabditis uteleia* | CSP31.g122.t1 |
| *Caenorhabditis uteleia* | CSP31.g17985.t1 |
| *Caenorhabditis uteleia* | CSP31.g18117.t1 |
| *Caenorhabditis sulstoni* | CSP32.g15412.t1 |
| *Caenorhabditis sulstoni* | CSP32.g342.t1 |
| *Caenorhabditis sulstoni* | CSP32.g4273.t1 |
| *Caenorhabditis sulstoni* | CSP32.g6536.t1 |
| *Caenorhabditis sulstoni* | CSP32.g8262.t1 |
| *Caenorhabditis quiockensis* | CSP38.g12453.t1 |
| *Caenorhabditis quiockensis* | CSP38.g1970.t1 |
| *Caenorhabditis quiockensis* | CSP38.g22150.t1 |
| *Caenorhabditis quiockensis* | CSP38.g4271.t1 |
| *Caenorhabditis quiockensis* | CSP38.g7811.t1 |
| *Caenorhabditis waitukubuli* | CSP39.g231.t1 |
| *Caenorhabditis waitukubuli* | CSP39.g25818.t1 |
| *Caenorhabditis waitukubuli* | CSP39.g8430.t1 |
| *Caenorhabditis waitukubuli* | CSP39.g9166.t1 |
| *Caenorhabditis tribulationis* | CSP40.g17956.t1 |
| *Caenorhabditis tribulationis* | CSP40.g24648.t1 |
| *Caenorhabditis tribulationis* | CSP40.g4891.t1 |
| *Caenorhabditis tribulationis* | CSP40.g7742.t1 |
| *Caenorhabditis tribulationis* | CSP40.g8381.t1 |
| *Caenorhabditis sinica* | Csp5_scaffold_00402.g10818.t1 |
| *Caenorhabditis sinica* | Csp5_scaffold_00758.g15180.t1 |
| *Caenorhabditis sinica* | Csp5_scaffold_00998.g17423.t1 |
| *Caenorhabditis sinica* | Csp5_scaffold_02322.g25969.t1 |
| *Trichuris suis* | D918_03995 |
| *Diploscapter coronatus* | DCO_007495 |
| *Diploscapter coronatus* | DCO_007496 |
| *Diploscapter coronatus* | DCO_007565 |
| *Diploscapter coronatus* | DCO_007566 |
| *Diploscapter coronatus* | DCO_008107 |
| *Diploscapter coronatus* | DCO_008108 |
| *Diploscapter coronatus* | DCO_008755 |
| *Diploscapter coronatus* | DCO_008756 |
| *Diploscapter coronatus* | DCO_011551 |
| *Diploscapter coronatus* | DCO_011552 |
| *Ditylenchus destructor* | Dd_05855 |
| *Ditylenchus destructor* | Dd_09592 |
| *Ditylenchus destructor* | Dd_09983 |
| *Dictyocaulus viviparus* | DICVIV_03779 |
| *Dracunculus medinensis* | DME_0000091801-mRNA-1 |
| *Dracunculus medinensis* | DME_0000646701-mRNA-1 |
| *Elaeophora elaphi* | EEL_0000617401-mRNA-1 |
| *Elaeophora elaphi* | EEL_0000947401-mRNA-1 |
| *Loa loa* | EN70_2393 |
| *Loa loa* | EN70_4364 |
| *Loa loa* | EN70_7180 |
| *Pristionchus entomophagus* | entomophagus-mkr-S16-5.37-mRNA-1 |
| *Pristionchus entomophagus* | entomophagus-mkr-S42-7.46-mRNA-1 |
| *Pristionchus entomophagus* | entomophagus-mkr-S486-1.83-mRNA-1 |
| *Pristionchus entomophagus* | entomophagus-mkr-S52-4.31-mRNA-1 |
| *Panagrolaimus es5* | ES5_v2.g13486.t1 |
| *Pristionchus exspectatus* | exspectatus-mkr-S_10-2.14-mRNA-1 |
| *Pristionchus exspectatus* | exspectatus-mkr-S_449-0.59-mRNA-1 |
| *Pristionchus exspectatus* | exspectatus-mkr-S_496-0.53-mRNA-1 |
| *Pristionchus exspectatus* | exspectatus-sn_msk-S_619-0.27-mRNA-1 |
| *Caenorhabditis elegans* | F11A5.10 |
| *Caenorhabditis elegans* | F25F8.2 |
| *Pristionchus fissidentatus* | fissidentatus-mkr-S152-2.42-mRNA-1 |
| *Pristionchus fissidentatus* | fissidentatus-mkr-S310-0.2-mRNA-1 |
| *Pristionchus fissidentatus* | fissidentatus-mkr-S310-1.11-mRNA-1 |
| *Pristionchus fissidentatus* | fissidentatus-sn_msk-S218-1.45-mRNA-1 |
| *Caenorhabditis remanei* | FL82_08070 |
| *Caenorhabditis remanei* | FL82_08370 |
| *Caenorhabditis remanei* | FL82_10926 |
| *Caenorhabditis remanei* | FL82_12334 |
| *Caenorhabditis remanei* | FL82_20361 |
| *Caenorhabditis latens* | FL83_03810 |
| *Caenorhabditis latens* | FL83_09497 |
| *Caenorhabditis latens* | FL83_12043 |
| *Globodera pallida* | GPLIN_000391800 |
| *Globodera rostochiensis* | GROS_g11465.t1 |
| *Globodera rostochiensis* | GROS_g13114.t1 |
| *Heterorhabditis bacteriophora* | Hba_18793 |
| *Haemonchus contortus* | HCON_00001030-00001 |
| *Haemonchus contortus* | HCON_00020000-00001 |
| *Haemonchus contortus* | HCON_00057900-00001 |
| *Haemonchus contortus* | HCON_00148840-00001 |
| *Haemonchus contortus* | HCON_00161180-00001 |
| *Heterodera glycines* | Hetgly.G000003670 |
| *Heterodera glycines* | Hetgly.G000010604 |
| *Halicephalobus mephisto* | HMEPH_00833-RA.p1 |
| *Halicephalobus mephisto* | HMEPH_02028-RA.p1 |
| *Halicephalobus mephisto* | HMEPH_03034-RA.p1 |
| *Halicephalobus mephisto* | HMEPH_03577-RA.p1 |
| *Haemonchus placei* | HPLM_0000029401-mRNA-1 |
| *Haemonchus placei* | HPLM_0001088901-mRNA-1 |
| *Haemonchus placei* | HPLM_0001206301-mRNA-1 |
| *Haemonchus placei* | HPLM_0001844101-mRNA-1 |
| *Heligmosomoides polygyrus* | HPOL_0000096601-mRNA-1 |
| *Heligmosomoides polygyrus* | HPOL_0000126101-mRNA-1 |
| *Heligmosomoides polygyrus* | HPOL_0000210301-mRNA-1 |
| *Heligmosomoides polygyrus* | HPOL_0001003101-mRNA-1 |
| *Heligmosomoides polygyrus* | HPOL_0001177601-mRNA-1 |
| *Pristionchus japonicus* | japonicus-mkr-S15-5.51-mRNA-1 |
| *Pristionchus japonicus* | japonicus-mkr-S3-16.49-mRNA-1 |
| *Pristionchus japonicus* | japonicus-mkr-S91-2.18-mRNA-1 |
| *Ditylenchus dipsaci* | jg1357 |
| *Ditylenchus dipsaci* | jg8839 |
| *Propanagrolaimus ju765* | JU765_v2.g14513.t1 |
| *Steinernema carpocapsae* | L596_010789 |
| *Steinernema carpocapsae* | L596_012353.1 |
| *Steinernema carpocapsae* | L596_013989 |
| *Steinernema carpocapsae* | L596_947553 |
| *Steinernema feltiae* | L889_g13345.t1 |
| *Steinernema feltiae* | L889_g16301.t1 |
| *Steinernema scapterisci* | L892_g12947.t1 |
| *Steinernema scapterisci* | L892_g17141.t1 |
| *Steinernema scapterisci* | L892_g31358.t1 |
| *Steinernema scapterisci* | L892_g4398.t1 |
| *Steinernema glaseri* | L893_g24687.t1 |
| *Steinernema glaseri* | L893_g32461.t1 |
| *Steinernema monticolum* | L898_g27172.t1 |
| *Steinernema monticolum* | L898_g3657.t1 |
| *Steinernema monticolum* | L898_g467.t1 |
| *Meloidogyne javanica* | M.Javanica_Scaff1049g012318 |
| *Meloidogyne javanica* | M.Javanica_Scaff12776g065608 |
| *Meloidogyne javanica* | M.Javanica_Scaff15752g073075 |
| *Meloidogyne javanica* | M.Javanica_Scaff18311g078500 |
| *Meloidogyne javanica* | M.Javanica_Scaff18732g079349 |
| *Meloidogyne javanica* | M.Javanica_Scaff1883g019056 |
| *Meloidogyne javanica* | M.Javanica_Scaff2051g020296 |
| *Meloidogyne javanica* | M.Javanica_Scaff6g000154 |
| *Meloidogyne javanica* | M.Javanica_Scaff909g011109 |
| *Wuchereria bancrofti* | maker-PairedContig_1362-snap-gene-0.13-mRNA-1 |
| *Wuchereria bancrofti* | maker-PairedContig_4818-snap-gene-0.10-mRNA-1 |
| *Wuchereria bancrofti* | maker-PairedContig_833-snap-gene-3.28-mRNA-1 |
| *Pristionchus maxplancki* | maxplancki-ag_msk-S276-1.25-mRNA-1 |
| *Pristionchus maxplancki* | maxplancki-mkr-S320-0.32-mRNA-1 |
| *Pristionchus maxplancki* | maxplancki-mkr-S695-0.3-mRNA-1 |
| *Pristionchus maxplancki* | maxplancki-mkr-S88-2.14-mRNA-1 |
| *Pristionchus mayeri* | mayeri-mkr-S38-1.54-mRNA-1 |
| *Pristionchus mayeri* | mayeri-sn_msk-S231-3.65-mRNA-1 |
| *Pristionchus mayeri* | mayeri-sn_msk-S70-0.40-mRNA-1 |
| *Mesorhabditis belari* | mbelari.g11282.t1 |
| *Mesorhabditis belari* | mbelari.g13813.t1 |
| *Mesorhabditis belari* | mbelari.g17487.t1 |
| *Mesorhabditis belari* | mbelari.g24023.t1 |
| *Mesorhabditis belari* | mbelari.g736.t1 |
| *Micoletzkya japonica* | MicoRS5524-mkr-S16-5.32-mRNA-1 |
| *Micoletzkya japonica* | MicoRS5524-mkr-S266-0.67-mRNA-1 |
| *Micoletzkya japonica* | MicoRS5524-mkr-S4-0.15-mRNA-1 |
| *Micoletzkya japonica* | MicoRS5524-mkr-S92-1.16-mRNA-1 |
| *Meloidogyne incognita* | Minc3s00160g06393 |
| *Meloidogyne incognita* | Minc3s00850g18087 |
| *Meloidogyne incognita* | Minc3s01194g21591 |
| *Meloidogyne incognita* | Minc3s03095g32755 |
| *Meloidogyne incognita* | Minc3s03785g34808 |
| *Meloidogyne incognita* | Minc3s04623g36686 |
| *Meloidogyne incognita* | Minc3s04683g36803 |
| *Halicephalobus mephisto* | MSTRG.15328.1.p1 |
| *Halicephalobus mephisto* | MSTRG.15328.2.p1 |
| *Halicephalobus mephisto* | MSTRG.15328.3.p1 |
| *Halicephalobus mephisto* | MSTRG.15328.4.p1 |
| *Halicephalobus mephisto* | MSTRG.15328.5.p1 |
| *Halicephalobus mephisto* | MSTRG.15328.7.p1 |
| *Halicephalobus mephisto* | MSTRG.7093.1.p1 |
| *Halicephalobus mephisto* | MSTRG.8361.1.p1 |
| *Acanthocheilonema viteae* | nAv.1.0.1.t04012-RA |
| *Acanthocheilonema viteae* | nAv.1.0.1.t10056-RA |
| *Nippostrongylus brasiliensis* | NBR_0001349101-mRNA-1 |
| *Nippostrongylus brasiliensis* | NBR_0001782501-mRNA-1 |
| *Nippostrongylus brasiliensis* | NBR_0001888601-mRNA-1 |
| *Dirofilaria immitis* | nDi.2.2.2.t00192 |
| *Dirofilaria immitis* | nDi.2.2.2.t07691 |
| *Necator americanus* | NECAME_06509 |
| *Necator americanus* | NECAME_16744 |
| *Necator americanus* | NECAME_16780 |
| *Litomosoides sigmodontis* | nLs.2.1.2.t00112-RA |
| *Litomosoides sigmodontis* | nLs.2.1.2.t03804-RA |
| *Meloidogyne graminicola* | NXFT01001786.1.4605_g |
| *Meloidogyne graminicola* | NXFT01001833.1.4739_g |
| *Meloidogyne graminicola* | NXFT01002921.1.7654_g |
| *Oesophagostomum dentatum* | OESDEN_10587 |
| *Onchocerca ochengi* | OOCN_0000005801-mRNA-1 |
| *Onchocerca ochengi* | OOCN_0000465001-mRNA-1 |
| *Onchocerca ochengi* | OOCN_0000505501-mRNA-1 |
| *Onchocerca ochengi* | OOCN_0000638401-mRNA-1 |
| *Oscheius tipulae* | OTIPU.nOt.2.0.1.t00171 |
| *Oscheius tipulae* | OTIPU.nOt.2.0.1.t05169 |
| *Oscheius tipulae* | OTIPU.nOt.2.0.1.t08848 |
| *Onchocerca volvulus* | OVOC10658 |
| *Onchocerca volvulus* | OVOC290 |
| *Onchocerca volvulus* | OVOC388a |
| *Onchocerca volvulus* | OVOC388c |
| *Onchocerca volvulus* | OVOC6009 |
| *Panagrellus redivivus* | Pan_g14961.t1 |
| *Panagrellus redivivus* | Pan_g17723.t1 |
| *Panagrellus redivivus* | Pan_g1951.t1 |
| *Panagrellus redivivus* | Pan_g20505.t1 |
| *Panagrellus redivivus* | Pan_g686.t1 |
| *Parapristionchus giblindavisi* | Parapristionchus-mkr-S_202-0.80-mRNA-1 |
| *Parapristionchus giblindavisi* | Parapristionchus-mkr-S_326-0.83-mRNA-1 |
| *Parapristionchus giblindavisi* | Parapristionchus-mkr-S_395-0.56-mRNA-1 |
| *Parascaris univalens* | PgB05_g069_t01 |
| *Parascaris univalens* | PgB06_g029_t01 |
| *Pristionchus pacificus* | PPA12815 |
| *Pristionchus pacificus* | PPA15833 |
| *Pristionchus pacificus* | PPA18552 |
| *Pristionchus pacificus* | PPA26611 |
| *Pristionchus pacificus* | PPA35955 |
| *Panagrolaimus ps1159* | PS1159_v2.g24106.t1 |
| *Panagrolaimus ps1159* | PS1159_v2.g9269.t1 |
| *Plectus sambesii* | PSAMB.scaffold1476size31015.g13330.t1 |
| *Plectus sambesii* | PSAMB.scaffold2268size24200.g17136.t1 |
| *Plectus sambesii* | PSAMB.scaffold4007size16052.g23134.t1 |
| *Plectus sambesii* | PSAMB.scaffold4365size14887.g24144.t1 |
| *Parastrongyloides trichosuri* | PTRK_0000480500.1 |
| *Parastrongyloides trichosuri* | PTRK_0000767100.1 |
| *Parastrongyloides trichosuri* | PTRK_0001406100.1 |
| *Parastrongyloides trichosuri* | PTRK_0001593100.1 |
| *Caenorhabditis elegans* | R11G10.1a |
| *Meloidogyne enterolobii* | scaffold10175_cov179.g13551 |
| *Meloidogyne enterolobii* | scaffold2007_cov180.g3397 |
| *Meloidogyne floridensis* | scf7180000423303.g10645 |
| *Setaria digitata* | sdigi.contig253.g6720.t1 |
| *Setaria digitata* | sdigi.contig42.g2702.t1 |
| *Syphacia muris* | SMUV_0000707701-mRNA-1 |
| *Syphacia muris* | SMUV_0000880401-mRNA-1 |
| *Wuchereria bancrofti* | snap_masked-PairedContig_4222-processed-gene-0.0-mRNA-1 |
| *Caenorhabditis inopinata* | Sp34_10080600.t1 |
| *Caenorhabditis inopinata* | Sp34_10287700.t1 |
| *Caenorhabditis inopinata* | Sp34_20261600.t1 |
| *Caenorhabditis inopinata* | Sp34_50160400.t1 |
| *Caenorhabditis inopinata* | Sp34_50194700.t1 |
| *Strongyloides papillosus* | SPAL_0000281900.1 |
| *Strongyloides papillosus* | SPAL_0000967700.1 |
| *Strongyloides papillosus* | SPAL_0001712600.1 |
| *Strongyloides ratti* | SRAE_0000037800 |
| *Strongyloides ratti* | SRAE_2000230900 |
| *Strongyloides ratti* | SRAE_2000376800 |
| *Strongyloides ratti* | SRAE_X000111500 |
| *Strongyloides stercoralis* | SSTP_0000778000.1 |
| *Strongyloides stercoralis* | SSTP_0000796200.1 |
| *Strongyloides stercoralis* | SSTP_0001177900.1 |
| *Strongyloides stercoralis* | SSTP_0001253100.1 |
| *Strongyloides venezuelensis* | SVE_0146400.1 |
| *Strongyloides venezuelensis* | SVE_0530300.1 |
| *Strongyloides venezuelensis* | SVE_0564500.1 |
| *Strongyloides venezuelensis* | SVE_1002500.1 |
| *Trichinella britovi* | T03_5966.1 |
| *Trichinella t6* | T06_10544.1 |
| *Trichinella papuae* | T10_7609.1 |
| *Trichinella zimbabwensis* | T11_17426.1 |
| *Trichinella pseudospiralis* | T4E_11705.1 |
| *Toxocara canis* | Tcan_14831.1 |
| *Toxocara canis* | Tcan_16332.1 |
| *Toxocara canis* | Tcan_16956.1 |
| *Thelazia callipaeda* | TCLT_0000779101-mRNA-1 |
| *Thelazia callipaeda* | TCLT_0000861301-mRNA-1 |
| *Thelazia callipaeda* | TCLT_0000924801-mRNA-1 |
| *Teladorsagia circumcincta* | TELCIR_03204 |
| *Teladorsagia circumcincta* | TELCIR_04975 |
| *Teladorsagia circumcincta* | TELCIR_05497 |
| *Meloidogyne arenaria* | tig00000344.g49440.t1 |
| *Meloidogyne arenaria* | tig00000502.g56256.t1 |
| *Meloidogyne arenaria* | tig00000818.g65392.t1 |
| *Meloidogyne arenaria* | tig00001475.g19227.t1 |
| *Meloidogyne arenaria* | tig00001624.g35071.t1 |
| *Meloidogyne arenaria* | tig00002126.g52460.t1 |
| *Meloidogyne arenaria* | tig00002139.g39721.t1 |
| *Trichuris trichiura* | TTRE_0000568501-mRNA-1 |
| *Diploscapter pachys* | WR25_04210A.1 |
| *Diploscapter pachys* | WR25_04210B.1 |
| *Diploscapter pachys* | WR25_04210C.1 |
| *Diploscapter pachys* | WR25_04210E.1 |
| *Diploscapter pachys* | WR25_04210H.1 |
| *Diploscapter pachys* | WR25_13557A.1 |
| *Diploscapter pachys* | WR25_13557B.1 |
| *Diploscapter pachys* | WR25_15342.1 |
| *Diploscapter pachys* | WR25_16134.1 |
| *Onchocerca flexuosa* | X798_00812 |
| *Onchocerca flexuosa* | X798_01724 |
| *Caenorhabditis elegans* | ZC317.3 |
