# Supplementary material for: Database of glutamate-gated chloride (GluCl) subunits across 125 nematode species: patterns of gene accretion and sequence diversification
Source: G3 (Bethesda). 2021 Dec 21;12(2):jkab438. doi: 10.1093/g3journal/jkab438 (PMC9210312; doi:10.1093/g3journal/jkab438)
Supplement: jkab438_Supplemental_Material_Legends [file jkab438_supplemental_material_legends.docx]

**Supplemental data**

**Figure S1.** Bar plot depicting observed and expected numbers of GluCl gene counts per Clade.

**Figure S2.** Correlation plot of GluCl gene counts versus BUSCO scores.

**Figure S3.** Distribution of BUSCO scores across the assemblies used to predict GluCl subunit genes.

**Figure S4.** Chromosomal location of the *glc-1*, *glc-2*, *glc-3*, *glc-4*, *avr-14*, and *avr-15* genes in *C. elegans* on chromosomes I, II, and V.

**Figure S5.** Bar plot depicting observed and expected numbers of GluCl gene counts parsed by lifestyle. The lifestyle were organized into animal parasitic, human parasitic, plant parasitic, insect parasitic, and free-living.

**Figure S6.** Sequence alignment of TM3 from multiple nematode species indicating diversity within a key residue involved in IVM binding.

**Table S1.** Species and genome assembly data used to predict GluCl genes

**Table S2.** Species and transcripts used in figure 2 phylogeny
